# Supplementary material for: Functionally Isolated Sarcoplasmic Reticulum in Cardiomyocytes: Experimental and Mathematical Models
Source: Bioengineering (Basel). 2025 Jun 9;12(6):627. doi: 10.3390/bioengineering12060627 (PMC12189891; doi:10.3390/bioengineering12060627)
Supplement: Supplementary file 1 [file bioengineering-12-00627-s001.zip › bioengineering-3648254-supplementary.pdf]

Functionally Isolated Sarcoplasmic Reticulum in Cardiomyocytes: Experimental and Mathematical Models

Diogo C. Soriano [1,2,*], Rosana A. Bassani [3] and José W. M. Bassani [1,3]

[1] Department of Electronics and Biomedical Engineering, School of Electrical and Computer Engineering, Universidade Estadual de Campinas (UNICAMP), Av. Albert Einstein 400, Campinas 13083-852, SP, Brazil; bassani@unicamp.br
[2] Center for Engineering, Modeling and Social Sciences, Universidade Federal do ABC (UFABC), Alameda da Universidade, s/n, São Bernardo do Campo 09606-045, SP, Brazil
[3] Brazilian Laboratory for Cellular Calcium Research (LabNECC), Center for Biomedical Engineering, Universidade Estadual de Campinas (UNICAMP), R. Alexander Fleming 163, Campinas 13083-881, SP, Brazil; rbassani@unicamp.br
* Correspondence: diogo.soriano@ufabc.edu.br

SUPPLEMENTARY MATERIAL

Incorporating Caffeine Model into the Bondarenko Framework

This supplementary material outlines the mathematical framework and modifications applied to incorporate the caffeine-induced calcium release mechanism into the model by Bondarenko et al. (2004).

Figure S1 presents a schematic overview of the **FISRM (functionally isolated sarcoplasmic reticulum model)**, integrating key assumptions from the original Bondarenko model and the proposed caffeine-induced Ca^{2+} dynamics, under the proposed conditions. In an extracellular medium nominally free of Na^+ and Ca^{2+} ($[Ca^{2+}]o \approx 0$, $[Na^+]o \approx 0$), and Ca^{2+} release from the sarcoplasmic reticulum (SR) triggered by caffeine, several membrane ion currents are inoperant, including those mediated by the Na^+/K^+ ATPase (I_{NaK}) and the Na^+/Ca^{2+} exchanger (I_{NaCa}), as well as the fast Na^+ current (I_{Na}), L-type Ca^{2+} current (I_{CaL}), and both Ca^{2+} and Na^+ background currents (I_{Cab}, I_{Nab}), so that the membrane potential is at its resting value. The sarcolemmal (SL) Ca^{2+} pump, as well as K^+ and Cl^- currents, remain active and are modeled as originally described in the Bondarenko formulation.

Intracellular Ca^{2+} fluxes are represented by uptake into the network SR (J_{up}) by the SR Ca^{2+}-ATPase (SERCA), release from the junctional SR (J_{rel}), transfer between the network and junctional SR (J_{tr}), passive leak from the SR (J_{leak}), transfer from the subspace to the

myoplasm (J_{xfer}) and binding to troponin C (J_{trpn}). The model also includes passive Ca^{2+} buffering by calmodulin in the cytosol and by calsequestrin within the SR.

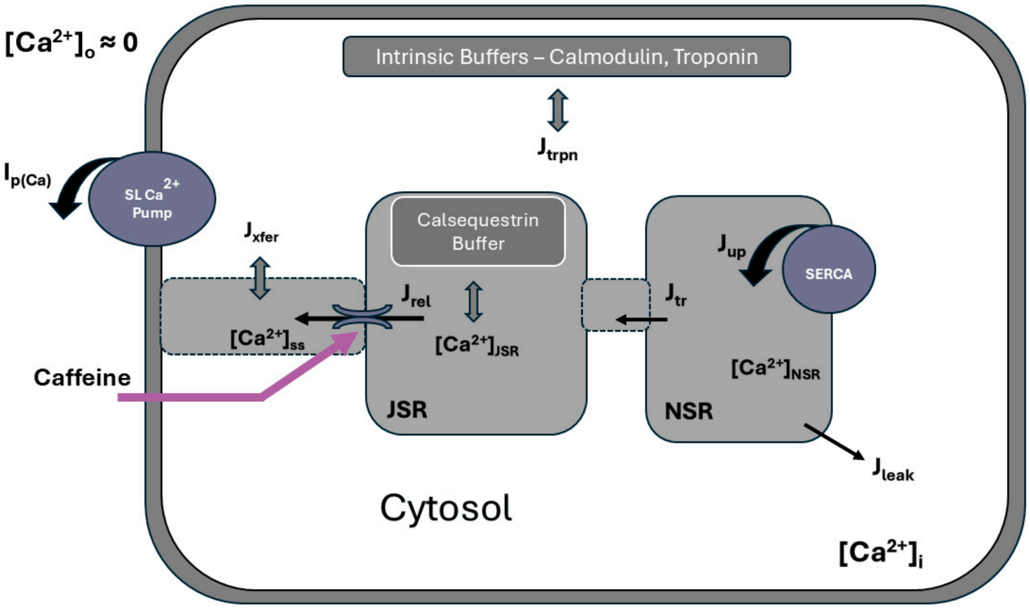

Figure S1. Schematic representation of the functional isolated sarcoplasmic reticulum model (FISRM) incorporating the mechanism of caffeine-induced calcium release. The diagram is based on the structure of the model by Bondarenko et al. (2004), modified to accommodate FISRM assumptions. Caffeine induces Ca^{2+} release (J_{rel}) from the junctional SR (JSR) by directly acting on SR Ca^{2+}release channels. Ca^{2+} fluxes include uptake into the network SR (NSR) via SR Ca^{2+}-ATPase (SERCA) (J_{up}), transfer between NSR and JSR (J_{tr}), leakage from the SR to the cytosol (J_{leak}), movement between the subspace and bulk cytosol (J_{xfer}) and buffering by cytosolic troponin C and calmodulin (J_{trpn}). The SL Ca^{2+} pump ($I_{p(Ca)}$), which is much less abundant than SERCA, is active. Other major sarcolemmal currents are inoperative under low $[Ca^{2+}]_o$ and $[Na^+]_o$ conditions.

In the following pages, only the core equations of the Bondarenko´s model that are relevant to the FISRM are indicated. The conditions, parameter alterations and key components are systematically outlined for the simulations performed.

Core Equations from the Bondarenko Model

Calcium Fluxes

1. **Uptake Flux (SERCA):** (Equation A13 in Bondarenko et al.)

$$J_{up} = \frac{V_{max,up} \cdot [Ca^{2+}]_i}{K_{m,up} + [Ca^{2+}]_i} \quad (1)$$

Where:

- $V_{max,up}$: Maximum calcium uptake rate ($\mu M/ms$)
- $K_{m,up}$: Michaelis-Menten constant for SERCA (μM)

2. **Release Flux (RyR):** (Equation A14 in Bondarenko et al.)

$$J_{rel} = v \cdot P_{RyR} \cdot (P_{O1} + P_{O2}) \cdot \left([Ca^{2+}]_{JSR} - [Ca^{2+}]_{SS}\right) \quad (2)$$

Where:

- v: Maximum total RyR permeability ($\mu M/ms$)
- P_{RyR}: RyR modulation factor
- P_{O1} and P_{O2}: Fractions of RyR in open states
- $[Ca^{2+}]_{JSR}$: Calcium concentration in junctional SR (μM)
- $[Ca^{2+}]_{SS}$: Calcium concentration in subsarcolemmal space (μM)

Dynamics of RyR States (Equations A18 and A20 in Bondarenko et al.)

The state equations for RyR behavior are considering fractions in the open state:

$$\frac{d(P_{O1})}{dt} = k_a^+ \cdot [Ca^{2+}]_{ss}^n \, P_{C1} - k_a^- \cdot P_{O1} - \cdots$$

$$\cdots k_b^+ \cdot [Ca^{2+}]_{ss}^m \, P_{O1} + k_b^- \cdot P_{O2} - k_c^+ \cdot P_{O1} + k_c^- \cdot P_{c2} \qquad (3)$$

$$\frac{d(P_{O2})}{dt} = k_b^+ \cdot [Ca^{2+}]_{ss}^m \, P_{O1} - k_b^- \cdot P_{O2} \qquad (4)$$

Where: k_x are transition rates

Caffeine Modulation Factor (G_{caff})

The transition rates of the RyR are modulated by a caffeine $G_{caff}(t)$ factor described by first order dynamics:

$$\frac{dG_{caff}(t)}{dt} = \frac{G_{caff}(\infty) - G_{caff}(t)}{\tau_{caff}} \qquad (5)$$

Where:

τ_{caff}: is the caffeine time constant (ms) set in 50 ms according to Su et al, 2003.

The new $k_a^{+\prime}$ and $k_c^{-\prime}$ transition rates for the RyR states are dependent on the caffeine modulation factor in the form:

$$k_a^{+\prime} = G_{caff}(t) \cdot k_a^+ \qquad (6)$$

$$k_c^{-\prime} = G_{caff}(t) \cdot k_c^{-} \qquad (7)$$

RyR Modulation Factor (P_{RyR}) (Equation A15 in Bondarenko et al.)

The original Bondarenko et al. equation for the modulation factor (A15) is preserved under default extracellular Ca^{2+} and Na^{+} conditions. Under $[Ca^{2+}]_o$ and $[Na^{+}]_o$ absence (Ty00 condition), the modulation factor follows first order dynamics in the form:

$$\frac{dP_{RyR}(t)}{dt} = \frac{P_{RyR}(\infty) - P_{RyR}(t)}{\tau_{RyR}} \qquad (8)$$

Where:

- $P_{RyR}(\infty)$: steady-state value for the RyR modulation factor sensible to caffeine. It is 0 in the absence of caffeine and 0.2542 for the first 20 ms of caffeine application
- τ_{RyR}: Time constant (ms) also sensible to caffeine. It is 75 ms in the absence of caffeine and 10 ms for the first 20 ms of caffeine application.

  The dynamics of P_{RyR} leads to bel-shape RyR flux release (J_{rel}) under caffeine presence.

Modified Simulation Conditions

Ty00 Condition (0 Extracellular Calcium and Sodium)

To simulate Ty00 we assumed: 1) first order P_{RyR} dynamics (equation 8); 2) $[Ca^{2+}]_o = 1\mu M, \quad [Na^{+}]_o = 1\mu M$ (residual concentration). Default values in the Bondarenko model are $[Ca^{2+}]_o = 1800\mu M$ and $[Na^{+}]_o = 140{,}000\mu M$, when $[Na^{+}]_o$ and $[Ca^{2+}]_o$ are present in the extracellular environment (TyN condition).

Caffeine Application

During caffeine application (first 20 ms):

- $P_{RyR}(\infty) = 0.2542$
- $\tau_{RyR} = 10$ ms

Outside this window:

- $P_{RyR}(\infty) = 0$
- $\tau_{RyR} = 75$ ms

Isoproterenol (ISO) Application

Under ISO conditions:

- g_{CaL} increased by 40%:

$$g_{CaL} = 1.4 \times 0.1729 = 0.2421 \text{ mS/μF}.$$

- $K_{m,up}$ decreased by 35%:

$$K_{m,up} = 0.65 \times 0.5 = 0.325 \text{ μM}.$$

- $P_{RyR}(\infty)$ changed to 0.1372.

tBQ Application

Under tBQ conditions:

- v_3 (SERCA uptake rate) reduced by 40%:

$$v_3 = 0.6 \times 0.45 = 0.27 \text{ μM/ms}.$$

- $P_{RyR,\infty}$ increased by 60%:

$$P_{RyR}(\infty) = 1.6 \times 0.2542 = 0.4067.$$

Table of Parameters

Parameter	Default Value	ISO Condition	tBQ Condition
g_{CaL} (mS/μF)	0.1729	0.2421 (+40%)	0.1729 (no change)
$K_{m,up}$ (μM)	0.5	0.325 (-35%)	0.5 (no change)
v_3 (μM/ms)	0.45	0.45 (no change)	0.27 (-40%)
$P_{RyR}(\infty)$	0	0.1372	0.4067 (+60%)

Table of Constants

Constant	Symbol	Default Value	Unit
Maximum RyR Permeability	v	3.2e-5	$\mu M/ms$
SERCA Michaelis Constant	$K_{m,up}$	0.5	μM
SERCA Uptake Rate	v_3	0.45	$\mu M/ms$
L-type Ca Current Conductance	g_{CaL}	0.1729	mS/μF
Total High-Affinity Buffer	$HTRPN_{tot}$	140	μM
Total Low-Affinity Buffer	$LTRPN_{tot}$	70	μM

Summary

This material focuses on the integration of our caffeine model with the Bondarenko framework, referencing equations from Bondarenko's appendix where applicable. Specific equations and parameter adjustments are highlighted to capture the experimental conditions, ensuring an accurate simulation of Ca^{2+} dynamics in the FISR model. These modifications provide a new framework for studying Ca^{2+} fluxes and RyR modulation under experimental and simulated conditions.

Code

Code for simulating the conditions shown in the paper can be found in the link:

https://github.com/dcsoriano/FISR-Model

Briefly, Figure 5 in the paper can be obtained through the code:

Sim_1_FISR_model_Exp_Protocol/Main_FISR_model_exp_protocol_Fig_5.m

Figure 8 (ISO Condition) can be obtained using the code:

Sim_2_FISR_ISO/Main_Adjust_Parameters_ISO_Condition_Fig_8.m

Figure 11 (tBQ Condition) can be obtained using the code:

Sim_3_FISR_tBQ/Main_test_varia_Vmax_caff_stim_tBQ_Fig_11.m
